# The ‘disenchantment’ of traditional acupuncturists in higher education

**DOI:** 10.1177/1363459321990725

**Published:** 2021-02-21

**Authors:** Assaf Givati, Shelley Berlinsky

**Affiliations:** King’s College London, UK; City College of Acupuncture, UK

**Keywords:** complementary and alternative medicine, narrative analysis, professions and professionalisation

## Abstract

Efforts of traditional acupuncturists in the UK to regulate their practice and standardise their training, led, from the mid-1990s, to the launch of acupuncture undergraduate programmes within, or validated by, universities. It appeared as if by so doing acupuncturists were on course to align themselves with ‘scientifically plausible’, state-regulated, allied health professionals, a remarkable development considering the marginality of acupuncture practice outside East Asia, and its paradigmatic tensions with biomedicine. But was it really to be? Based on in-depth interviews with higher education acupuncture educators and an analysis of educational documents published by the leading professional body, we explore the way in which this paradigmatic tension is negotiated within a framework that is dominated by biomedicine. By critically revisiting sociology of professions and anti-colonial analysis, we examine an over two decades long journey of acupuncture educators in academic institutions in the UK. Based on this analysis, we point at some of the challenges that acupuncturists faced in higher education that may have restricted the academic legitimisation of acupuncture and that left them in a position of academic marginality and greater exposure to scrutiny, leading to their academic and mainstreaming ‘disillusionment’. At the same time, by positioning themselves as ‘professional academics’ within higher education institutions and demonstrating professionalism, acupuncture educators were able to demonstrate academic and professional ‘credibility’ and therefore distance themselves from the continuous scrutiny over their ‘biomedical fragility’.

## Introduction

This research study explores the rise and the decline of acupuncture training programmes in higher education institutions (HEIs) in the UK during acupuncturists’ efforts to obtain ‘legitimate’ academic status within a system that is dominated by biomedicine. Central to this investigation are acupuncture educators’ attempts to bridge the gap between institutional demands to standardise and formalise their esoteric knowledge, including the ‘biomedical adjustment’ of their programmes, while maintaining the authenticity of their ancient theories and practices. This has been a dual challenge of operating within an unaccommodating knowledge framework while trying to secure an institutional academic standing to a discipline which its public reputation in Britain has been, for many years, fuelled by ‘curiosity, mysticism, fear, loathing, desperation, or simply a sense of novelty (Bivins, 2010: 175)’.

The emergence of Chinese medicine (CM) outside East Asia since the 1970s’ and its ‘nesting’ and its proliferation in Europe, the Americas and Australasia (EAA), can be characterised as a process of gradual yet striking transition. Over the past five decades, it shifted from a position of ‘biomedical otherness’ that is based on ‘holistic’, anti-reductionist narratives and claims that mirrored the counter-culture critique of biomedicine^1^ (Bates, 2002; Cant and Sharma, 1999; Porter, 2002; Rosenberg, 1998), to the adoption of ‘mainstreaming’ strategies and the efforts to ‘normalise’ and ‘legitimate’ practice (Baer, 2009; Barnes, 2003). Traditional acupuncture, one of the branches of CM and the centre of our study, is the most known and widespread practice of traditional medicine worldwide (CAMDOC Alliance, 2010).

During the 1970s, acupuncture in EAA emerged in popularity alongside other complementary and alternative medicine (CAM) as part of the medical counter-culture. Since the 1980s, acupuncturists were typically engaged in mobilising their practice from the fringes towards mainstream medical care by adopting the same *professionalisation* strategies as ‘conventional’ health care practitioners (Almeida and Gabe, 2016; Barnes, 2003; Bivins, 2010; Cant, 2009; Givati and Hatton, 2015; Saks, 2003; Welsh et al., 2004). These professional strategies included the formalisation of their knowledge base by establishing standardised training programmes, some of which, since the mid-1990s, took place in Higher Education Institutions (HEIs), in countries like Australia (Baer, 2015; Brosnan et al., 2016; Zheng, 2014), the UK (Cant and Sharma, 1999; Givati and Hatton, 2015; Saks, 2001), and the US (Barnes, 2003; Flesch, 2013). Against this backdrop, the aim of this study is to explore acupuncture educators’ perceived experience of over two decades in HEIs, and the way paradigmatic and academic tensions with biomedicine and the academic institutions were negotiated within a framework that is designed to accommodate medical sciences.

## Analytical framework and analytical challenges

Sociologists’ reference to the academisation of acupuncture training programmes has been dominated by credentialism and professionalisation framework. Mainly, it is argued, acupuncturists tried to obtain external legitimacy through ‘mainstreaming’ strategies, including the codification of their esoteric knowledge by developing accreditation mechanism for their training programmes and schools (Almeida, Siegel and De Barros, 2018; Baer, 2015; Barnes, 2003; Cant and Sharma, 1999; Givati and Hatton, 2015; Ijaz et al., 2016; Saks, 1999; Shuval and Averbuch, 2012).

In the context of professionalisation, the formalising of expert knowledge in HEIs legitimises higher societal status as it enables the standardisation and the accreditation of professionally aspiring groups according to the accepted ‘mainstream’ paradigm (Collins, 1979; Larson, 1979; Witz, 1995). In the case of traditional acupuncture, the accepted paradigm with which it is aligned is biomedicine (Cant and Sharma, 1999), which presents what can be described as a ‘paradigmatic mismatch’. Therefore, while the formalised acupuncture curriculum has been typically infused with biomedical knowledge (Saks, 2003; Welsh et al., 2004), the nature of teaching this knowledge as part of acupuncture education, however, is an ‘unchartered territory’, which has been largely left unexplored (Brosnan, 2017; Ijaz et al., 2016; Hollenberg and Muzzin, 2010).

According to Freidson (1988) and Larson (1979), at the heart of professionalisation is the interplay between power and knowledge that evolves in relation to the construction of a formal knowledge-base while obtaining market power (Witz, 1995). Therefore, professionalisation is seen as a process of translating ‘one order of scarce resources – special knowledge and skills – into another – social and economic reward’ (Larson, 1977, xvi, xvii in Witz, 1995). A key dimension of professional projects is the negotiation of cognitive exclusiveness over a relatively abstract knowledge that is subject to practical application (Larson, 1979). In the case of the medical profession, the establishment of a professional market involved the standardisation of professional services and of providers, the elimination of competitors and state’s support for the medical profession’s monopoly over medical education and competency (Saks, 1999; Witz, 1995), including the formalisation and standardisation of medical practice and training.

Obtaining formal credentials in HEIs allows professional organisations to determine which qualifications should be attained by its members to perform a range of occupational tasks, placing certificate-bearing practitioners in a position of ‘experts’. By protecting their occupational jurisdiction through credentials, practitioners are able to obtain status and economic gains and exercise discretion (Abbott, 1988; Murphy, 1988), thus differentiating members of the professional groups from ‘externals’ who do not obtain the same certificates (Collins, 1979; Parkin, 1979).

### Critique of the professionalisation framework

Despite its prominence in the sociological analysis of CAM, the professionalisation analytical framework of CM outside East Asia attracted critique over some conceptual limitations. First, it is not very clear how the epistemic tension between CM and biomedicine is managed as part of teaching itself, how it influences the position of acupuncture in HE, and whether or not it really does bring to societal or economic gains. Moreover, the meaning of the terms ‘alignment’ with biomedicine or ‘infusion’ of biomedical knowledge in the context of academic CM programmes remains rather obscure. Furthermore, since traditional acupuncture is not a unified nor ‘static’ medical system but dynamic practice tradition that draws on diverse schools of thought, it is not clear how this plurality affects the accreditation and standardisation of teaching programmes.

The work of the sociologists Jamous and Peloille’s (1970) is useful in demonstrating the gap between CM and biomedicine in HEIs. It suggests that professional work is a mixture of *technical (T) activities* and *indeterminate (I) judgement*, which is expressed as a ratio between the two (I/T ratio). The *indeterminate* nature of practice is the emphasis on *secrecy, exclusivity* and *experience* that are based on practitioners’ personal and social qualities. The *technical* knowledge is *regulated, codified*, and *standardised* through rules, and is the knowledge that is *transparent* and externally scrutinised. The latter reflects the *scientific, biomedical* basis of medical knowledge and its supposed construction around reductionism, quantifiability and objectivity (Traynor, 2009).

In the case of traditional acupuncture, this model demonstrates some of the challenges of teaching acupuncture programmes in HEIs. like some other CAM (Hirschkorn, 2006), acupuncturists often argue that their practice relies on ‘indeterminate, situated knowledge gained through experience: a form of craft knowledge inherently resistant to codification’ (Clarke et al., 2004, p.330), or what Jamous and Peloille characterised as high I/T ratio. At the same time, maintaining high degree of indeterminacy alludes to clinical ‘vagueness’ and lack of transparency in making clinical judgements, which does not correspond with the professional aspiration for trustworthiness and public credibility that was aspired for through the academisation of their training programmes. Moreover, as Jamous and Peloille proposed, being studied in academic institutions includes a process of routinising, documenting and exposing the previously ‘hidden’ esoteric knowledge, which meant that acupuncturists were making themselves more exposed to external scrutiny and criticism (Givati and Hatton, 2015).

### An anti-colonial critique

Another critique of the professionalisation framework is over its lack of engagement with the ‘biomedical otherness’ of indigenous medical systems outside the geo-cultural setting from which these systems emerged (Cant, 2020; Fadlon, 2004; Hollenberg and Muzzin, 2010). While many popular CAM therapies and practices emerged alongside scientific medicine that followed the scientific revolution in Europe and North America from the 1700s onwards^2^ (Bates, 2002), both Chinese medicine and Ayurveda are *thousands*-years-old *indigenous Asian* medical systems, and as argued by Cant (2020):

While the focus on professionalisation tells us much about occupational strategies, territorial and jurisdictional battles, it does not reveal the deep, cultural, knowledge wars that are also enacted in, and through, medical pluralism (p. 45).

During the 1960s and 1970s, alternative therapies re-emerged as part of the medical counter-culture that has been dominated by Marxist, feminist and post-colonial movements (Boston Women’s Health Collective, 1973; Brown, 1976; Ehrenreich, 1978; Illich, 1975), and by the medicalisation thesis (Szasz, 2007; Zola, 1972). The reductionist account of biomedicine invited approaches that claimed to be more ‘holistic’ and more personalised and which pay more attention to the individual patient and her/his voice (Bates, 2002; Goldstein, 1999). Fanon’s post-colonial critique (Fanon, 1952, and then 1961) contributed to the medical counter-culture perspective by discussing the importance of nationhood in former colonies, the ongoing colonisation of mental health and the view of non-Europeans as ‘others’ which is rooted in biomedical power and control. It argued that the way by which colonial biomedical doctors ignored indigenous practices and perceptions of health, disease, and medical practices, contributed to the oppression of indigenous communities and were therefore part of the colonial project.

Central to the anti-colonial literature was the ‘imagined’ and socially constructed nature of the representation of indigenous knowledge (Anderson, 1983) and the way by which cultural illustrations and imaging of ‘the East’ and the vision of ‘East’ and ‘West’ are both assumed and exaggerated and are infused by hierarchical difference in which ‘Western otherness’ is equated with subjugation (Said, 1979). Within this framework, Anderson (2002), and Anderson and Adams (2008), questioned the positioning of indigenous medicines in North America, raising questions over the social and political boundaries of the distribution, the mobilisation and the adaptation of this ‘other (non-biomedical) knowledge’, and how it is standardised or transformed in relation to the dominant, biomedical framework. As Adams (2002) argues, the effort to bring together two medical systems involves several contestations including how does one medical system deliberate between different claims to truth offered by the other, and what determines the boundaries of scientific legitimacy.

In their anticolonial discussion of CAM, Hollenberg and Muzzin (2010) pointed at the privileged and unquestioned position of the biomedical paradigm in settings whereby CAM and biomedicine are ‘integrated’. They suggest that in such settings indigenous knowledge is being appropriated, assimilated, and often devaluated - and even dismissed as non-objective and non-empirical. At the same time, they argue, biomedically-dominated frameworks and institutions cannot reconcile non-materialist concepts of vitality that are central to indigenous medical systems. We therefore return to the question of how this epistemic gap is negotiated in acupuncture programmes in HE.

### The evolution of Chinese medicine under biomedical hegemony

Chinese medicine as a complete medical system integrates several branches of practice. It includes acupuncture and moxibustion, herbal medicine, *tui na* (a form of manual therapy), diet, and *tai chi* and *qi gong* which are perhaps best described as a meditative exercise to control the flow of *qi* (Scheid, 2002). During its over 2000 years-long history CM has been dynamic in nature, continuously shaped in relation to both local and global political and societal circumstances and historical events (Barnes, 2005; Bivins, 2010; Hsu, 2008; Napolitano and Flores, 2003; Scheid, 2002), constructed and enacted by practitioners and their users, educators and their students, policy-makers and trend-setters.

The concept ‘Chinese’ medicine was formed out of the need to differentiate between the medical practice in China for over 2000 years, from that of ‘the west’, as biomedicine was increasingly growing in popularity and dominance worldwide and in China (Andrews, 2015). This has been a clash between different philosophical, cultural, and scientific approaches. The arrival of modern biomedicine to China during the second half of the 19th century and the first half of the 20th century, was paralleled by the declining support for CM. From 1911, the republic of China tried to establish a modern state medical system based on biomedicine while, at the same time, discouraging the practice of CM (Hsu, 2008; Taylor, 2005).

However, after 1949, with the communist rule in People’s Republic of China (PRC), CM became an integral part of Mao’s ‘new China’ vision with the aim of creating an integrated *zhongyi* (Chinese medicine) as and *xiyi* (Western medicine) (Scheid, 2002; Tang, 2018).^3^ During this time CM underwent a process of institutionalisation, secularisation and ‘streamlining’ (Barnes, 2003; Hsu, 2008; Scheid, 2002; Taylor, 2005) through which ancient theoretical texts and practices were removed as it was deemed ‘superstitious’ (*mixin*) or religious in nature, and in order to enable the standardisation of this vast knowledge so that it is more *compatible* with ‘Western thinking’ and with biomedicine (Tang, 2018).

This institutionalisation project drew a line between ‘China of the old’ and the new communist China, but it was also pragmatic in nature. It was arguably designed to promote the ‘Western appeal’ of CM, while, at the same time, make the knowledge of CM cruder and more palatable so that it was easier to learn and apply in the many poor and remote areas in China that had no access to medical treatment (Cluett and Parker, 2017; Taylor, 2005). The result of this project was the standardisation of esoteric knowledge in the form of the *Traditional Chinese Medicine* (TCM) which, upon the removal of spiritual and religious esoteric texts, allowed for greater communication with, and assimilation of, biomedical concepts (Hsu, 2008). Variations of CM have also developed in neighbouring countries and, over time, in Europe and the US by individuals who travelled to, or worked, in East Asia, and based on their own interpretations of historical texts revived the popularity of CM in these regions (Stollberg, 2007).

The growing popularity, reputation and political domination of biomedicine since the 19^th^ century had major influence on the teaching and practice of acupuncture. While many biomedical practitioners in EAA adopted acupuncture techniques as part of their therapeutic arsenal, CM practitioners in East Asia embraced biomedical techniques and interventions to enrich the practice of acupuncture (Hsu, 2002). Similarly, traditional acupuncturists in the UK (Givati, 2015) and in the US (Barnes, 2005; Flesch, 2013) commonly adopt biomedical concepts to re-package CM concepts or use the findings of acupuncture-supportive biomedical research to enhance the public credibility of their practice (Givati and Hatton, 2015).

## Methodology

### Data collection and ethical approval

This qualitative study is based on two data sets: (a) between June 2015 and April 2016 we conducted in-depth, qualitative interviews with 10 traditional acupuncture educators, and (b) we conducted documentary analysis of undergraduate university-validated acupuncture courses’ syllabi, as well as educational documents published by the main professional body, the British Acupuncture Council (BAcC), and its accreditation arm. Ethical application was submitted, reviewed and obtained favourable opinion from the host university’s Research Ethics Committee in June 2015 (reference SFEC-2015-040).

### In depth interviews

Ten in-depth interviews were conducted with acupuncture educators (see table 1) with lecturing experience in university based (*N* = 5) or university validated (*N* = 5) acupuncture programmes in England. All ten participants, six women and four men, had substantial practice experience of between 11 to 38 years, averaging 24 years in practice. Practitioners were approached via their academic and/or professional email address. Sampling was purposive in the first place. Acupuncture educators in London and South of England (five out of the seven undergraduate programmes operating in the UK at the time of the study) were approached on the base of the programmes’ institutional staff list. A study information sheet was included in the original email and informed consent form was signed prior to interviews.

Interviews lasted between 40 and 90 minutes and were guided by a protocol of broad discussion subjects (see ‘data analysis’ section) that has been generated in relation to our analytical framework and the research questions. All interviews were recorded and transcribed verbatim by a professional transcribing agency. Interviewees identifiable information has not been included.

**Table 1.** Interviewees’ profile.

Interviewee number	Role in acupuncture education
INT1	Programme lead, university-based BSc (Hons) Acupuncture.
INT2	Senior Lecturer, university-based BSc (Hons) Acupuncture.
INT3	Previously Senior Lecturer in university-based BSc (Hons) and MSc acupuncture programmes. Currently the programme lead of university validated, private school BSc (Hons) Acupuncture.
INT4	Previously SL, BSc (Hons) Acupuncture university validated programme taught by a private school. Currently an SL in a BAcC accredited, non-university validated acupuncture programme.
INT5	Programme lead, university-based BSc (Hons) Acupuncture.
INT6	School manager and Senior Lecturer in university validated, private schools (Hons) acupuncture programme.
INT7	Previously school manager and SL, BSc (Hons) Acupuncture university validated programme taught in a private school. Currently an SL in a BAcC accredited, non-university validated acupuncture programme.
INT8	Previously the school manager of university-validated, private school BSc (Hons) Acupuncture programme. Currently the programme lead of non-university validated acupuncture programme.
INT9	Previously Senior lecturer at university validated, private school BSc (Hons) Acupuncture programme. Currently the programme lead of non-university validated acupuncture programme.
INT10	Senior lecturer at university-based BSc (Hons) Acupuncture.

### Data analysis

Data analysis was conducted according to qualitative content analysis (QCA), a method used to systematically describe the meanings of qualitative data (Mayring, 2004; Schreier, 2014). The method of QCA identifies key categories from the available research literature and from theoretical models and then turns to the data to consider how these categories are shaped and informed by participants’ narratives. While in-depth interviews are designed to promote interviewee’s’ self-reflection and rich storytelling, the process of QCA provides a systematic and pragmatic approach to manage the data. The first stage of the data analysis was to read through the text and reduce material that is strikingly unrelated to the research question. The next stage included the organisation of the text according to pre-defined ‘contextual categories’, which are developed from the theoretical models informing the investigation and against which the data is continuously considered. As part of the interview itself, participants were asked to first describe their acupuncture practice and teaching biography and reflect on the following contextual categories:

■ the evolution of the acupuncture practice and the acupuncture educational landscape in the UK since they qualified as practitioners,
■ challenges to the formalising of acupuncture education,
■ the role of HEIs in acupuncture training,
■ the development of acupuncture knowledge and practice within the university framework,
■ the process of negotiating CM theory alongside biomedical concepts and scientific research as part of university validates programmes,
■ the challenge of assessing difficult-to-standardise practice skills, for example pulse diagnosis, tongue diagnosis^4^ or manipulating *qi*, within standard university benchmark,
■ the challenge of teaching acupuncture alongside other academic and professional disciplines.

These contextual categories only served to frame participants’ narratives and ensure that textual analysis corresponds with the theoretical framework. They were then re-visited as data emerges, and as emerging interview-narratives elicit new research-related concepts and ‘leads’, then the categories are modified accordingly, to maintain the flexible and inherently *inductive character* of QCA.

Thematic analysis itself had several layers: first, quotes from participants’ narratives were organised under the analytical units. When this process is completed, the text under each unit was analysed for sub-themes. As that data has been organised into contextual categories, themes and sub-themes, it was easier to consider the ‘internal story’ within narratives. In order to enhance the likelihood of analytical generalisation, textual material is analysed systematically to the point of saturation of sub-themes.

### Documentary analysis

As suggested by Bowen (2009), documents can include data that provides both background information and historical context within which the research participants operate. In the case of acupuncture education and training, formal accreditation and standardisation documents demonstrated the strategies and discourses used by the professional and educational leadership in their efforts to manage ideological and practice-related tensions between biomedicine and acupuncture as part of their training programmes. The following documents were reviewed:

a) British Acupuncture Accreditation Board, Accreditation Handbook, 2010
b) BAcC Standards of Education and Training for Acupuncture (SETA), 2011
c) BAcC Standards of Practice for Acupuncture (SPA), 2009
d) Courses’ syllabus of all BAcC accredited, university validated training programmes which were available through the intuitions’ websites.

These documents were analysed too according to qualitative content analysis in relation to the following analytical units:

- the ‘traditional’ and biomedical content of expert knowledge being taught,
- the application of academic benchmark as part of the acupuncture/CM programme,
- the ‘infusion’ of biomedical knowledge including scientific investigation and research methods;
- academic and professional assessment and the examination of acupuncture practice skills.

First, the documents were read through to reduce material that is strikingly unrelated to the research question, after which the remaining text was organised in columns under analytical units to allow for the clustering of text in relation to key categories, followed by the re-reading and organisation of categories according to sub-themes which captures the ‘internal story’ that illuminates the analytical units.

## Findings

Our acupuncture educators’ narratives evolved around three key themes: (a) the ‘normalisation’ of acupuncture in HEIs and its degree of academic acceptance alongside mainstream professional and academic disciplines; (b) the nature of alignment with biomedicine and the ‘academic credibility’ of acupuncture; and (c) the tension between academic standardisation and maintaining the authenticity and the plurality of practice traditions.

### Coming out of isolation? Acupuncture educators seeking academic acceptance

The first acupuncture training courses were established in the UK during the 1960’s and 1970’s (Uddin, 2008, in Moir, 2020). The process of standardizing acupuncture education started in 1980 with the unification of disparate acupuncture associations that represented diverse practice traditions, and the establishment of the British Acupuncture Accreditation Board (BAAB) a decade later (Saks, 1999). The teaching of traditional acupuncture in British universities since 1996 inspired a growing sense of optimism that acupuncture^5^ is moving closer to obtain mainstream status. Despite question marks over the degree of subordination to biomedicine, the growing number of undergraduate acupuncture programmes between 1995 and 2010 appeared to suggest that acupuncturists were gaining ‘legitimate’ academic status (Givati, 2015). At its peak, in 2010, there were fourteen undergraduate acupuncture programmes in the UK, of which eight were taught within universities and another six were delivered in private acupuncture schools that had obtained university accreditation (Givati and Hatton, 2015). There was a sense amongst acupuncture educators that their programmes gained sustained academic status.

The motivation behind acupuncture educators’ academic endeavour was varied. INT1, programme lead of one *BSc (Hons) Acupuncture*, was actively involved with the academisation of CM in the UK since the mid-1990s. During her interview she described the decision to leave the ‘sheltered yet isolated’ position that characterised acupuncture practice in the UK during the 1970s and 1980s prior to the launch of HE programmes:

We thought that Chinese medicine will be better placed in universities. There will be better student support, better teaching and learning resources, a library, online resources, registration system, better facilities. But perhaps most importantly was that we felt a need to come out of the isolated position we were in and meet other academic and professional disciplines.

Indeed, during the following two decades an academic department for CM was established at that university, including several undergraduate and postgraduate academic pathways. However, over time, as INT1 suggests, it became apparent that the isolation of Chinese medicine continued inside university walls:

[When we joined the university in 1995] we thought it is better to be “a little fish in a big pond”, yet we were worried that we will end up in a broom cupboard at the end of the corridor. … In fact, at the School of Life Sciences, we were never accepted. It always felt like “them” and “us”, even though our students did very well on the science units such as anatomy and physiology, which they shared with students of “mainstream” professions.

INT2, a senior lecturer on another undergraduate acupuncture programme, described similar frustration:

Interviewer: *Did you have any contacts, discussions or interaction with other departments and lecturers in your faculty, like biomedical science for example?*

INT2: Not really, let’s say that it was a case of “arranged marriage” …

Since 2010, acupuncture programmes in HEIs had a turn to the worse. Acupuncturists’ determined effort to obtain state regulation was first boosted by a House of Lords Select Committee on Science and Technology’s Report on Complementary and Alternative Medicine, that in 2000 recommended that acupuncturists strive for statutory regulation under the Health Act 1999 (House of Lords’ Science and Technology committee, 2000). However, a decade later, the Secretary of State for Health announced that acupuncture will remain self-regulated (The Secretary of State for Health, 16 Feb 2011: Column 84WS), which meant that almost anyone can practice acupuncture regardless of the level of training or qualification. In the years that followed, several acupuncture programmes in HE closed, leaving only four active undergraduate programmes of which one stopped recruiting new students (British Acupuncture Council, 2020).

Furthermore, as Jamous and Peloille’s (1970) analytical framework predicts, by standardising their knowledge in HEIs acupuncturists were running the risk of ‘exposing’ their knowledge to external scrutiny, and therefore to attacks from competitors and ‘opponents’. Along with other CAM academic programmes, undergraduate acupuncture programmes were under persistent and organised scrutiny by a group of scientists, academics and journalists who opposed the teaching in universities of the ‘biomedically implausible’, new academic discipline (Brosnan and Cribb, 2019; Givati and Hatton, 2015).

As in Caldwell’s (2017) boundary work analysis of homeopathy in HE, such attacks can be seen as an effort to demarcate ‘true science’ from ‘non-science’ (Hess, 1993; Gieryn, 1983) in order to disrepute the ‘less authoritative’ and ‘non-scientific’ alternative practitioners - now in HEIs. Amongst the boundary work strategies enacted towards acupuncturists were the scrutinising of scientific evidence for acupuncture (Ernst, 2017; Colquhoun and Novella, 2013) and of universities teaching CAM programmes (Colquhoun, 2007; Corbyn, 2008), as well as the contrasting of acupuncture’s mechanism of action with the logic of science to demonstrate its ‘irrationality’ (Derbyshire, 2013).

### ‘Integration, ‘hybridity” or ‘domestication”? the nature of the alignment with biomedicine and enhancing the academic credibility of acupuncture programmes

The academisation of acupuncture required adaptation to the HE landscape on several levels. Barnes (2003), Brosnan and Cribb (2019) and Flesch (2013), discussed the tension between the professional and the academic spheres of Chinese medicine. This tension, between professional practice and the requirement to adhere to the formal academic benchmarks, characterises the academisation of any professional group, but in the case of acupuncture it is being intensified by the highly indeterminate nature of esoteric knowledge and the heavy reliance on practitioners’ discretion to make clinical interpretations based on experience, intuition and ‘artistic’ skills.

One strategy of acupuncture educators of addressing this challenge was by demonstrating academic and scientific ‘belonging’ in host academic departments: acupuncture programmes in HEIs were taught in academic faculties including *Science and Technology, Health & Social Care* and *Social and Life Science*, alongside ‘mainstream’ academic and professional disciplines such as, among others, nursing, biomedical science, psychology, radiography, physiotherapy, occupational therapy and social work. At the same time, acupuncture educators were increasingly obtaining higher academic degrees and Doctorates in disciplines like education, pharmacology, immunology and others.

The terms ‘alignment’ with biomedicine (Cant and Sharma, 1999) and biomedical ‘infusion’ (Welsh et al., 2004) that were used to describe CAM therapies’ professional strategy of ‘conforming’ to biomedical domination in order to enhance mainstream status, elude to the inclusion of biomedical subjects such as biology, physiology, pharmacology and scientific research in the formal acupuncture programmes. In practice, rather than some form of integration of two medical paradigms, academic acupuncture programmes largely maintain separation between the teaching of Chinese medicine and the teaching of biomedical knowledge. In one programme students do attend a module titled “Integrative Practice”, although the main objective of the module is ensuring students’ awareness of clinical circumstances that call for the utilisation of biomedical knowledge and tests (such as “red flags”), and awareness of the limitations of acupuncture practice and ensuring “safe practice”. Therefore, rather than adopting ‘biomedicalised’ version of acupuncture (as in the case of ‘medical’ or ‘Western’ acupuncture), students are asked to engage with two medical systems while maintaining the integrity of both paradigms. For example, M1, a course lead, argues that…

Although we moved into university, we teach “proper” traditional acupuncture including “proper” practice-based teaching, *heat, heart* [traditional CM theoretical concepts] and all that. What the university gave us was “the head”, the education, and it opened us to a wider world. It exposed us to research, it stimulated us, and it took us out of our insularity.

Furthermore, a review of the *BAcC Standards of Education and Training for Acupuncture* (2011, p.10), suggests that the biomedical knowledge is utilised *to complement* CM and provide it with mainstream context, such as to enable students to identify medical ‘red flags’; be able to consider a patient’s (bio)medical history including previous diagnosis and treatment; be able to communicate with biomedical practitioners; and engage with scientific research to inform practice. Arguably, in spite of the dominant position of biomedicine in HEIs, this is not quite the paradigmatic integration that is often eluded to, and as Gale (2014) warns, ‘integration, therefore, runs the risk of becoming a conceptual dead end without more selective and critical deployment’ (p. 811).

Rather, the process of biomedical alignment does not lead to the ‘bending’ or curtailing of CM theories, and biomedicine is utilised pragmatically to address facets of practice which are required to enhance the societal acceptance of acupuncture and provide it with greater ‘credibility’. Instead, undergraduate acupuncture programmes include the teaching, in parallel, of three ‘kinds’ of academic modules: ‘pure’ traditional acupuncture theory and practice modules; ‘pure’ biomedical science modules such as anatomy and physiology, pathophysiology, pharmacology and scientific research method; and ‘peripheral’ to the medical practice knowledge modules that ‘contextualise’ practice such as “interprofessional practice”, “running a clinic” and “sociology of health”. This academic landscape better corresponds with the notion of *domestication*, as biomedical knowledge is included in the acupuncture curriculum so that ‘the foreign is rendered familiar and palatable to local taste’ (Fadlon, 2004: 71).

For example, during her interview, INT9 discussed the research dissertation module that she coordinates, during which students are expected to review research evidence on the effectiveness of acupuncture for a range of medical conditions while following research models such as *PICO* (*Population, Intervention, Control, Outcome*), designed to support the development of a clinical research question, and *CASP* (Critical Appraisal Skills Programme), a checklist for the critical appraisal of research publications. Following these ‘standard’ research protocols provides acupuncture programmes with a sort of ‘academic credibility’. However, according to INT9, students’ effort to conduct ‘hybrid projects’ designed to consider the conceptual relationship between CM and biomedical theories such as ‘does research in Western medicine support the aetiologies of multiple sclerosis in traditional Chinese medicine’, or, ‘comparing the explanatory models of Western medicine and TCM for the pathophysiology of rhinitis’, resulted in a sort of ‘conceptual dead end’ and often in frustrating student experience as a result.

### The challenge of academic standardisation and plurality of practice styles

One of the challenges to the standardisation of acupuncture teaching programmes was the diversity of practice traditions and the question of ‘what kind of acupuncture’ should be taught as part of the standard acupuncture curriculum. While teaching programmes in the UK typically included the standardised *TCM*, there are other practice traditions that are influenced by ‘pre-TCM’ texts and theories, as well as more recent interpretations of such medical texts by charismatic teachers who set up the first CM schools in the UK, including the likes of J.R. Worsley and Dick Van Buren, who taught their own learning and narration of CM theory and practice.

INT10 was a member of the BAcC education committee that during the first half of the 1990s developed an educational regulatory framework for acupuncture education. In her interview she reflected on the difficulty to unify the diverse acupuncture schools and agree on a standard curriculum. She recalls the meeting:

So, on one side [of the room] we had the medical acupuncture group, and they weren’t interested in Chinese medicine theory at all. Then, on another side [of the room], there was the Chinese medicine group. And I include in that group traditional Chinese medicine *with a capital T*, traditional Chinese medicine *with a small T*, you know, practitioners who practice according to key principles of Chinese medicine including *Yin Yang* theory, *Ba Gang* and *Wu Xing*^6^… And then, on another side [of the room] we had the core ‘*five elements’* practitioners… [. .] The issue of practice styles is a huge problem…

Eventually, a core TCM curriculum was agreed, aligned with the professional body’s *Standards of Practice for Acupuncture (SPA)* document that all accredited colleges had to follow, while, in addition, each programme was able to teach its own unique approach. This resulted in a diversity of programmes and a plurality of practice styles. For example, while some schools contended that ‘TCM’ already includes the theory and practice of the Five Elements, another school taught Worsley’s unique version of *Five Elements acupuncture*, while yet another school was renowned for teaching Van Buren’s *Stems and Branches* acupuncture. Crucially, the position of the regulatory body, the BAcC, was that:

The BAcC has long embraced this plurality [of practice styles] under the heading “unity in diversity” and sees the variety of [acupuncture] approaches as the mark of a healthy profession.

Nevertheless, the question of what should and should not be included within the defined scope of acupuncture professional knowledge remained a thorny issue. Like some of interviewees, INT2, a senior lecturer in one of the university programs, argues that acupuncture schools are still strongly influenced by the individual subjective interpretation - or ‘fantasies’ as she calls it, of European ‘charismatic teachers’ who founded some of the schools. During the 1970s and 1980s, INT2 underwent acupuncture training with some of the most renowned acupuncture teachers in Europe and in the US. Later, she travelled to China and interviewed some of the few remaining pre-revolution CM masters prior to its standardisation into *TCM*:

In the college where I started acupuncture training, I found that it was very hierarchical, and it was based on cult personality [of the college founder] rather than what Chinese medicine is really about, according to my own reading of historical texts and philosophy. Unfortunately, because they [the teachers] wrote some of the [modern] textbooks, they are still the ones that students learn from and they are still very influential. So really, a lot of the teaching is based on these kinds of fantasies of the founders.

Although TCM acupuncture is included by all training programmes in the UK, some of the other practice traditions are arguably less ‘biomedically-compatible’. For example, *Worsley’s five elements* acupuncture heavily relies on the experiential and often the sensual and intuitive qualities of the practitioner. The text below, from the BAcC website, describes the *five elements* theory as…

…. .a way of understanding the energy of the body as a microcosm which reflects the macrocosm, the world around us. The elements, which are Fire, Earth, Metal, Water and Wood represent, for example, the different qualities which each season brings to the year but are also thought to be a way of understanding all phenomena. […] when Five Element practitioners describe someone as a Fire person or a Metal person, they are often paying homage to the work of J.R.Worsley who pioneered what he called the ‘causative factor’ as a way of understanding the person and treating them. In his view, in each person one of the elements was the one which was primary one out of balance, and treating this part of the system would restore overall balance.

Another programme which has even wider ‘biomedical distance’ teaches *stems and branches* acupuncture. The philosophical underpinning of this approach stems from the ancient text, the *Inner Canon of the Yellow Emperor*, which is considered the doctrinal source of CM, and is over 2000 years old. In this practice style, diagnosis includes the patient’s date and time of birth, according which practitioners often draw a detailed map of the patient’s’ energetic balance/imbalance. The following description is taken from the school’s website:

Stems and Branches theory is about cycles, transformations and interactions of *qi* that affects a person both internally and externally. The ancient Chinese observed the cycles of nature: day and night and the changing seasons. They recognised that the natural rhythms of nature underlie the most fundamental aspects of our lives.

One course leader argues that the wide biomedical gap inherent in practice styles such as *stems and branches* makes it difficult to defend the academic credibility of acupuncture within universities:

INT5: I am very happy for any sceptic to come along and I am very happy to take them on debate on what we teach. But I am not so happy to take them on if what they put forward is something like *stems and branches*. It is much harder. [In the case of TCM] I can make recalls to [biomedical] knowledge on endorphins, brain scans, and so on. In the case of *stems and branches*, it is not possible to justify it.

It is possible to argue that traditional acupuncturists identified a strategy of immersing themselves in higher education while maintaining the authenticity of their practice during the formalisation of their educational structures. At the same time, in the eyes of ‘the scrutinisers’ of acupuncture in HEIs, such a wide practice knowledge-base, reflects lack of theoretical cohesion and the ‘biomedical gap’ inherent in some of the practice styles and their standardisation-resistant nature portraits a picture of an ‘unworthy academic discipline’.

## Concluding remarks

In this study we explored acupuncture educator’s journey in HE in the UK since the mid-1990s, including the challenges that they faced as a medical system that is based on philosophical foundation and theories different to biomedicine, yet is taught within a mainstream institution; the strategies that they enacted to become accepted academic discipline; and their aspirations, achievements and disappointments. We located our participants’ narratives in relation to some of the premises and limitations of the professionalisation framework: the way by which the epistemic tension between CM and biomedicine has been negotiated as part of the formalisation of acupuncture in HEIs and as part of the argued ‘alignment’ or ‘infusion’ of biomedicine into the acupuncture curriculum; the unquestioned position of the biomedical paradigm in mainstream institutions and the question whether CM knowledge is being integrated, hybridised or domesticated in HEIs; the challenge of standardisation versus maintaining the authenticity of practice and plurality of practice styles (Barnes, 2003; Cant 2020; Moir, 2020); and the challenge of the ‘indeterminate nature’ of practice and its adaptation to a standardisation framework (Clarke et al., 2004; Givati and Hatton, 2015).

Acupuncture educators’ hopes that the provision of their programmes in HE alongside mainstream academic and professional disciplines would enhance their professional and public credibility and status, or that the academic departments would embrace acupuncture and provide them with sheltered position from scrutiny over their ‘irrational’ theories, have not been fully materialised. While acupuncture educators in the UK immersed themselves in academic institutions and academic culture, the biomedical gap inherent in Chinese medicine has not been narrowed by merely the inclusion of biomedical teaching modules within acupuncture programmes. The effort to maintain ‘authenticity’ of practice, by adopting flexible standardisation strategy to enable the teaching and plurality of diverse practice traditions, may have diluted the formalisation of practice and the potential of narrowing institutional and biomedical gaps.

Either way, the drop in number of students and in academic programmes, the ongoing hostility and the sense of ‘mainstream [academic] marginality’ (Cant, 2009), that is, seemingly obtaining mainstream status as an academic discipline, but without really being embraced or respected, and having to fend off ongoing scrutiny, eventually eroded acupuncture educators’ enthusiasm and hopes. Instead, there was a sense of weariness about our participants’ narratives and a realisation that the prospect of obtaining mainstream status, for now, is not within reach. Yet, the disillusionment from the ‘grand’ professionalisation aspirations has been replaced by pragmatism. Acupuncture educators seemed to have shifted their attention to academic *professionalism*, demonstrating academic professional practice, carefully following educational and administrative protocols, procedures and tasks while maintaining their academic programmes and while obtaining academic degrees and certificates. Acupuncturists’ journey in higher education can also be viewed as a process of sobering and of ‘professional maturity’. After all, INT6 argues,

…acupuncture is miles better taught now than it was in any time in the history of this country, that’s for sure. The students are much better qualified and certainly, if someone had told me, back in the 1970s, that I’d now be the Dean of a college running a BSc in acupuncture then…well…I would have bet big money against that!

## Notes

1. Why do we use the term *biomedicine*? Modern western scientific medicine is often called ‘biomedical’ because of its overarching tendency to explain health in biological terms. Furthermore, in its ‘cosmopolitan’ sense, biomedicine is a healing tradition that is found all over the world and has broad support from governments and societies in individual states, but also globally, through organisations such as the WHO. And while other codified healing systems such as Chinese medicine and Ayurveda exist and may be growing in popularity, biomedicine obtained greater status, legitimacy and state support and is therefore the commonly used frame of reference in discussing the status of other healing systems.

2. Homeopathy (late 1700s), osteopathy (1870s), naturopathy (1700s), chiropractic (mid 1890s), medical herbalism, reflexology (1910s), are all examples for popular CAM that originated from Europe and North America during the past 250 years.

3. Chinese medicine emerged as part of ancient Chinese culture and philosophical roots which ‘reinforced the doctrine of the mean common to Confucianism, Taoism, and Buddhism (Chan, 1963: 246, in Quah, 2003)’.The term ‘traditional’ acupuncture is used in order to *differentiate* acupuncture practice-styles that stem from Chinese medicine, from ‘western’ or ‘medical’ or acupuncture which is the use of the needling technique but according to biomedical explanatory models that are completely ‘divorced’ from CM theory (see for example White, 2009).

4. Diagnosis in Traditional Chinese Medicine (TCM) is conducted according to four methods: visual inspection of the face, skin and particularly the tongue which reflects the internal organs; auscultation; palpation - particularly the wrist pulse; and inquiry.

5. While acupuncture is a practice-branch of (the wider) Chinese medicine, most undergraduate programmes in HEIs in the UK award(ed) degrees in acupuncture. Nevertheless, depending on the context, and since acupuncture practice in nested within the philosophy and principles of Chinese medicine, we refer interchangeably to ‘acupuncture programmes’ as well as ‘the teaching of CM’.

6. *Ba Gang* is the term which refers to diagnostic parameters in Chinese medicine theory. *Wu Xing* are the ‘five phases’ or ‘five elements’, a theory that conceptualises interactions and relationships between phenomena, and is central explanatory model also in Chinese medicine.
